# Light-Emitting Porphyrin Derivative Obtained from a Subproduct of the Cashew Nut Shell Liquid: A Promising Material for OLED Applications

**DOI:** 10.3390/ma12071063

**Published:** 2019-04-01

**Authors:** Nayane Maria de Amorim Lima, Harold José Camargo Avila, Cleber Fabiano do Nascimento Marchiori, Samuel Gondim Sampaio, João Paulo Ferreira Mota, Viviane Gomes Pereira Ribeiro, Claudenilson da Silva Clemente, Giuseppe Mele, Marco Cremona, Selma Elaine Mazzetto

**Affiliations:** 1 Laboratory of Products and Process Technology (LPT), Organic and Inorganic Chemistry Department, Federal University of Ceara (UFC), Campus do Pici, Fortaleza-CE 60440-900, Brazil; nayaneal@yahoo.com.br (N.M.d.A.L.); sam.gs@hotmail.com (S.G.S.); jpfmpro@gmail.com (J.P.F.M.); claudenilsonsc@gmail.com (C.d.S.C.); selma@ufc.br (S.E.M.); 2 Molecular Optoelectronic Laboratory (LOEM), Physics Department, Pontifical Catholic University of Rio de Janeiro (PUC-Rio), Rio de Janeiro-RJ 22451-900, Brazil; hjca1986@gmail.com; 3 Materials Theory Division, Department of Physics and Astronomy, Uppsala University, Box 534, SE-75121 Uppsala, Sweden; c.marchiori@physics.uu.se; 4 Institute of Exact and Natural Sciences - ICEN, University of International Integration of Afro-Brazilian Lusophony, Redenção-CE 62790-000, Brazil; vivianegpribeiro@live.com; 5 Department of Innovation Engineering, University of Salento, Via Arnesano, 73100 Lecce, Italy; giuseppe.mele@unisalento.it

**Keywords:** cashew nut shell liquid, porphyrins, photoluminescence, electroluminescence, OLEDs

## Abstract

In this work, the *meso*-tetra[4-(2-(3-n-pentadecylphenoxy)ethoxy]phenylporphyrin (H_2_P), obtained from the cashew nut shell liquid (CNSL), and its zinc (ZnP) and copper (CuP) metallic complexes, were applied as emitting layers in organic light emitting diodes (OLEDs). These compounds were characterized via optical and electrochemical analysis and the electroluminescent properties of the device have been studied. We performed a cyclic voltammetry analysis to determine the Highest Occupied Molecular Orbital (HOMO) and Lowest Unoccupied Molecular Orbital (LUMO) energy levels for the porphyrins, in order to select the proper materials to assemble the device. H_2_P and ZnP presented fluorescence emission band in the red region, from 601 nm to 718 nm. Moreover, we verified that the introduction of bulky substituents hinders the π–π stacking, favoring the emission in the film. In addition, the strongest emitter, ZnP, presented a threshold voltage of 4 V and the maximum irradiance of 10 μW cm^−2^ with a current density (J) of 15 mA cm^−2^ at 10 V. The CuP complex showed to be a favorable material for the design of OLEDs in the infrared. These results suggest that the porphyrins derived from a renewable source, such as CNSL, is a promising material to be used in organic optoelectronic devices such as OLEDs.

## 1. Introduction

In the last few decades, organic light-emitting diodes (OLEDs) have attracted interest due to the several advantages like flexible and full-color devices, wide-viewing angle, lightness, transparency, and low power consumption [1,2,3,4]. OLEDs are spread in several fields such as imaging, lighting, automotive, transportation, communication, agriculture, and medicine. These devices have been currently commercialized in TVs, tablets, computers, and smartphones [1,2,3,4,5].

An efficient OLED is obtained through a multi-layer arrangement used between the electrodes (anode and metallic cathode) and the emitting material layer (EML). Additional layers are composed of electron or hole injection layers (EIL, HIL), electron or hole transport layers (ETL, HTL), and in some cases, electron and/or hole blocking layers (EBL, HBL), which is applied to confine the charges within the EML [6,7]. For practical applications of colored displays, the three primary colors (blue, green, and red) are required [8,9].

Several studies highlight the optimal efficiency of polymer devices [10], polycyclic aromatic [11], and carbazole derivatives [12,13] as blue-and-green emitters. For red-emitting materials, porphyrins derivatives have received considerable attention for their saturated red emission, strong absorption, high stability, good film-forming properties, and higher efficiency of energy transfer and electron transport [8,9,14,15].

The chemical stability and the possibility of tailoring their optical properties make porphyrins promising candidates for active materials in optoelectronic devices, since the optical, photophysical, and electrochemical properties can be modified via the insertion of peripheral substituents on the ring and as well as metallic atoms on the porphyrins’ cores [15].

Nowadays, sustainability is becoming an important issue in device fabrication. A good way to make the process more environmentally friendly is to use low-cost renewable sources and simpler architecture (fewer layers) in the device assembling, as well as the use of common/abundant materials that provide the same efficiency [5].

In this context, cashew nut shell liquid (CNSL) was used as a natural alternative to build up the porphyrin structure. CNSL is a renewable and abundant source of phenolic compounds, obtained as a subproduct of the cashew nut industry (*Anacardium occidentale* L.) [16,17,18]. The industrial CNSL is mainly composed by cardanol (about 65%), which presents interesting chemical characteristics and allows diverse functionalization and applications [19,20,21,22].

Based on these considerations, and those in previous works from our group regarding the preparation of *meso*-porphyrins [18,21,23,24,25,26], we report the synthesis of cardanol-derivate porphyrins with respect to its application as an emitting layer in OLEDs. The photophysical and electrochemical properties of the porphyrins are investigated in solution and in thin film, and we also investigate the use of these compounds as emitters in simple OLED devices through electroluminescence analysis. These compounds showed a strong absorption in the visible region and a consequently pronounced photoemission in the red with a characteristic well-defined for porphyrins.

## 2. Materials and Methods

### 2.1. General

All reagents and solvents were purchased from Sigma-Aldrich (St. Louis, MO, USA), Synth (Diadema, Brazil), and Vetec Quimica (Rio de Janeiro, Brazil) and used as received. The solvents used were: hexane (C_6_H_14_), dichloromethane (CH_2_Cl_2_), chloroform (CHCl_3_), *N,N*-dimethylmethanamide (DMF), and ethanol (C_2_H_6_O). The chemical reagents used in this work were: pyrrole, boron trifluoride diethyl etherate (BF_3_·OEt_2_), 2,3-dichlro-5,6-dicyano-1,4-benzoquinone (DDQ), zinc acetate dihydrate (Zn(CH_3_COO)_2_·2H_2_O), and copper acetate dihydrate (Cu(CH_3_COO)_2_·2H_2_O). Poly(3,4-ethylenedioxythiophene)-poly(styrenesulfonate) (PEDOT:PSS) and 1,3,5-Tris(1-phenyl-1Hbenzimidazol-2-yl)benzene (TPBi) were purchased from Clevios (Heraeus, Hanau, Germany) and Lumtec (New Taipei City, Taiwan), respectively. Absorption and emission spectra of the solutions were measured in dichloromethane at room temperature with an Agilent Cary 60 UV-Vis spectrophotometer (Agilent, Santa Clara, CA, USA) and Shimadzu RF-6000 fluorimeter (Shimadzu, Kyoto, Japan), respectively. ^1^H-Nuclear Magnetic Resonance (NMR) and ^13^C-NMR spectra were obtained in deuterated chloroform (CDCl_3_) using an NMR Bruker Avance DPX 300 spectrometer (Billerica, MA, USA) operating at 300 (^1^H) and 75 (^13^C) megahertz (MHz). Mass spectra (MS) were analyzed on a Bruker Microflex LT (MaldiTOF) (Billerica, MA, USA) using alphacyano, and dichloromethane as a matrix and 5% TFA as mobile phase. Elemental analysis (CHN): the percentage composition of the samples was analyzed on a PerkinElmer 2400 Series II (Waltham, MA, USA). The X-ray diffraction (XRD) analysis was performed in an X-ray powder diffractometer Xpert Pro MPD (Panalytical) (Panalytical, Almelo, the Netherlands) using Bragg–Brentano geometry in the range of 5°–120° with a rate of 1° min^−1^. CoKα radiation (λ = 1.7889 Å) was used and the tube operated at 40 kV and 30 mA. The cyclic voltammetry (CV) was performed on an Ivium Compact Stat using glass carbon as working electrode, a platinum wire as the auxiliary electrode, and Ag/Ag^+^ as the reference electrode. The procedure was performed at room temperature under a nitrogen atmosphere at a range of −2 V to 2.0 V with a step of 50 mV s^−1^ and standardized for the redox couple ferrocenium/ferrocene (Fc^+^/Fc). The UV-Vis and photoluminescence (PL) spectra of the porphyrins were performed on thin films (30 nm) grown via spin coating. Tetrahydrofuran (THF) solutions of porphyrins were prepared with concentration of 4 mg/mL and the films were deposited at 900 rpm for 60 s onto quartz substrates. The deposited films were dried at 70 °C to remove residual solvent. The PL spectra of the thin films were recorded at room temperature (298 K) on a Photon Technology International (PTI) fluorescence spectrophotometer (Photon Technology International, Birmingham, NJ, USA).

### 2.2. Synthesis of the H_2_P, ZnP, and CuP porphyrins from Cardanol

**Scheme 1:**
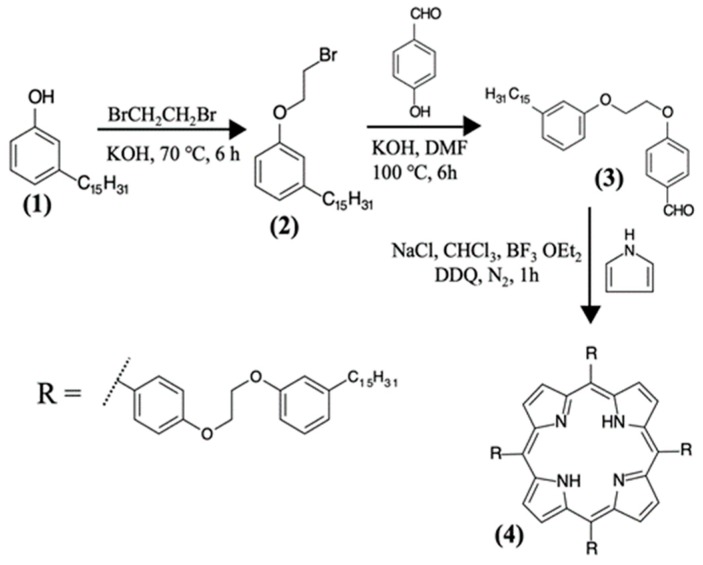
Synthesis of porphyrin H_2_P from cardanol.

The free-base *meso*-tetra[4-(2-(3-*n*-pentadecylphenoxy)ethoxy]phenyl porphyrin (H_2_P) from cardanol was prepared according to the procedure previously described in literature [18,23]. Despite the synthesis being already published, we highlight some relevant aspects here. The procedure followed three steps (Scheme 1). In the first step, the cardanol (13.16 mmol) (1) reacted with 1,2-dibromoethane (174.00 mmol) using KOH (39.49 mmol) as a base to give the (1-(2-bromoethoxy)-3-pentadecylbenzene) (2). The system was maintained at 70 °C and stirring for 6 h. The reaction mixture was treated with 50 mL of distilled water and filtered in a funnel with a paper filter. The obtained solid was purified through recrystallization as described in the methodology of Ribeiro et al. [18]. The product was obtained as a white solid and a reaction yield of 86% (4.6 g). In the second step, the synthesis of the 4-[2-(3-n-pendacylphenoxy)-ethoxy]benzaldehyde (3) was obtained via reaction of the compound 2 (7.30 mmol) and 4-hydroxybenzaldehyde (10.90 mmol) using KOH (23.30 mmol) as a base in 50 mL of DMF. The system was stirred at 100 °C for 6 h, then purified via recrystallization [18]. Finally, a white solid was obtained as a product with a yield of 56% (1.8 g).

The third phase involved the synthesis of 5,10,15,20-tetra-[4-(2-(3-pentadecyl)phenoxy)ethoxy]phenylporphyrin (4), which was obtained by reacting the compound (**3**) (2.21 mmol) with pyrrole (2.21 mmol) and NaCl (55.25 mmol) in 50 mL CHCl_3_ (stabilized with 0.8% ethanol) and degassed for 10 min with N_2_. Then, BF_3_OEt_2_ (0.72 mmol) was added. After 10 min, DDQ (1.64 mmol) was added and the mixture was stirred at room temperature for 1 h in an inert atmosphere. The reaction mixture was treated with 100 mL of a mixture of DMF/ethanol (7:3 *v*/*v*) under vigorous stirring. Then, the product was filtered and then purified via chromatography in a silica gel column with dichloromethane as the eluent. The final product was obtained as a purple solid with 26% (300 mg) reaction yield.

**Scheme 2:**
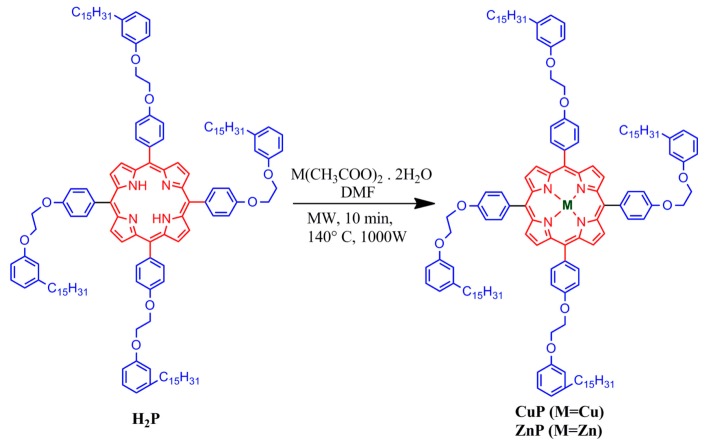
Synthesis of metalloporphyrins.

The synthesis of the derivatives of copper (CuP) and zinc (ZnP) was adapted from Attanasi et al. [27]. H_2_P (1 g, 0.50 mmol) was mixed with acetate metal salts X(CH_3_COO)_2_2H_2_O (X = Zn or Cu) (5 mmol) in DMF (20 mL) and the reaction mixture was refluxed under microwave irradiation for 10 min at 140 °C and the power was set at 1000 W (Scheme 2). After cooling to 25 °C, chloroform was added, and the mixture was washed with water. The chloroform layer was dried over Na_2_SO_4_, filtered, and concentrated under reduced pressure. The crude products were purified using silica gel column chromatography eluted with dichloromethane/hexane (7:3 *v*/*v*), and the complexes ZnP and CuP were collected in the first fraction eluted as purple with 87% (90 mg) and red with 93% (95 mg) solids, respectively, and characterized using NMR (Figures S1–S6), UV-visible spectroscopy and mass spectrometry (Figures S7–S9) and XRD analyses (Figure S10).

**H_2_P**: (26% yield). ^1^H NMR (300 MHz, CDCl_3_, δ ppm): −2.73 (s, 2H, N−H), 0.90 (t, 9H, *J* = 6.5 Hz, CH_3_), 1.28 (s, 72H, CH_2_–(CH_2_)_12_–CH_3_), 1.67 (m, 6H, Ph−CH_2_−CH_2_), 2.64 (t, 6H, *J* = 8.0 Hz, Ph−CH_2_), 4.51 (d, 12H, *J* = 4.5 Hz, O–(CH_2_)_2_−O), 6.85–8.12 (28H, Ph−H), 8.89 (s, 8H, H-β pyrrolic). ^13^C NMR (75 MHz, CDCl_3_, δ ppm): 14.30 (CH_3_), 22.89 (CH_2_–CH_3_), 29.56-32.13 (CH_2_−(CH_2_)_12_−CH_3_), 36.31 (Ph–CH_2_), 66.79 (O−(CH_2_)_2_−O), 111.91–158.99 (C-aromatic). MS (MALDI-TOF): *m/z*: for C_113_H_144_N_4_O_7_[M+], observed: 2000.0000; required: 2000.9224. Elemental analysis (CNH) for C_113_H_144_N_4_O_7_ (Found: C 81.29, N 3.65, H 8.76%; required: C 81.25, N 3.35, H 8.69%).

**CuP**: (yield: 93%). ^1^H NMR (300 MHz, CDCl_3_, δ ppm): 0.88 (t, 9H, *J* = 6.0 Hz, CH_3_), 1.27 (s, 72H, CH_2_−(CH_2_)_12_−CH_3_), 1.65 (m, 6H, Ph−CH_2_−CH_2_), 2.63 (t, 6H, Ph−CH_2_), 4.46 (d, 12H, O−(CH_2_)_2_−O), 6.86 (6H, H-aromatic). ^13^C NMR (75 MHz, CDCl_3_, δ ppm) 14.56 (CH_3_), 23.15 (CH_2_−CH_3_), 29.81–31.88 (CH_2_–(CH_2_)_12_−CH_2_), 36.55 (Ph−CH_2_), 66.45 (O–(CH_2_)_2_−O), 111.61–158.70 (C-aromatic). MS (MALDI-TOF): m/z: for C_113_H_142_N_4_O_7_Cu [M+], observed: 2062.0000; required: 2062.4526. Elemental analysis (CNH) for C_113_H_142_N_4_O_7_Cu (Found: C 78.28, N 3.47, H 8.41%; required: C 78.37, N 3.23, H 8.26%).

**ZnP**: (yield: 87%). ^1^H NMR (300 MHz, CDCl_3_, δ ppm) 0.88 (t, 9H, *J* = 6.0 Hz, CH_3_), 1.27 (s, 72H, CH_2_−(CH_2_)_12_−CH_3_), 1.68 (m, 6H, Ph−CH_2_−CH_2_), 2.63 (t, 6H, Ph−CH_2_), 4.54 (d, 12H, O−(CH_2_)_2_−O), 6.86–8.13 (28H, H-aromatic), 8.89 (s, 8H, H-β pyrrolic); ^13^C NMR (75 MHz, CDCl_3_, δ ppm) 14.10 (CH_3_), 22.68 (CH_2_−CH_3_), 29.36–31.93 (CH_2_–(CH_2_)_12_−CH_2_), 36.10 (Ph−CH_2_), 66.45 (O–(CH_2_)_2_−O), 111.73–150.53 (C-aromatic). MS (MALDI-TOF): m/z: for C_113_H_142_N_4_O_7_Zn [M+], observed: 2064.0000; required: 2064.2966; Elemental analysis (CNH) for C_113_H_142_N_4_O_7_Zn (Found: C 78.28, N 3.47, H 8.41%; required: C 78.37, N 3.23, H 8.26%).

### 2.3. Fabrication and Characterization of the OLEDs

The structure of devices consists in tri-layer architecture constructed onto ITO (indium tin oxide) coated glass substrates with a sheet resistance of 15 Ω square^−1^ (anode), which were cleaned via ultrasonication using an industrial detergent solution for 10 min, followed by consecutive ultrasonication in deionized water for 10 min until the detergent elimination. Finally, the substrates were ultrasonicated in acetone for 15 min and isopropyl alcohol for additional 15 min.

The PEDOT:PSS, used as hole transporting layer, was deposited using spin coating at 2000 rpm for 60 s and a thermal treatment at 100 °C. The emitting layer, i.e., the porphyrins (H_2_P, ZnP, and CuP), were dissolved in tetrahydrofuran (THF) 4 mg mL^−1^ and the films were deposited via spin coating (900 rpm for 60 s). The electron transporting layer, TPBi, as well as LiF and Al films, were successively deposited via thermal deposition in a Leybold deposition system (Leybold, Cologne, Germany) integrated in a MBraun glove-box (MBraun, Garching, Germany). The deposition chamber base pressure was 6.7 × 10^−4^ Pa. The deposition rates for organic was in the range of 0.1 nm s^−1^, the LiF was 0.01 nm s^−^1 and 0.2 nm s^−1^ for the aluminum cathode. The layer thickness was accurately controlled through a quartz crystal monitor (INFICON) (Inficon, Balzers, Liechtenstein) during the deposition and the thickness value was successively confirmed by a profilometer measurements. The OLEDs’ active areas were about 4 mm^2^ and the device were operated with a forward-bias voltage.

Electroluminescence (EL) spectra were obtained using a Photon Technology International (PTI) fluorescence spectrophotometer (Photon Technology International, Birmingham, NJ, USA) while EL brightness was measured with a calibrated radiometer/photometer (United Detector Technology, Hawthorne, CA, USA) from United Detector Technology (UDT-350). The current–voltage (J–V) characteristics were simultaneously measured with the EL spectra.

## 3. Results and Discussion

### 3.1. XRD Analysis, Absorption, and Photoluminescence Properties

The XRD analysis of the compounds H_2_P, CuP, and ZnP were performed and the shapes of the diffraction patterns were typical of polycrystalline powders, as shown in Figure S10.

**Figure 1:**
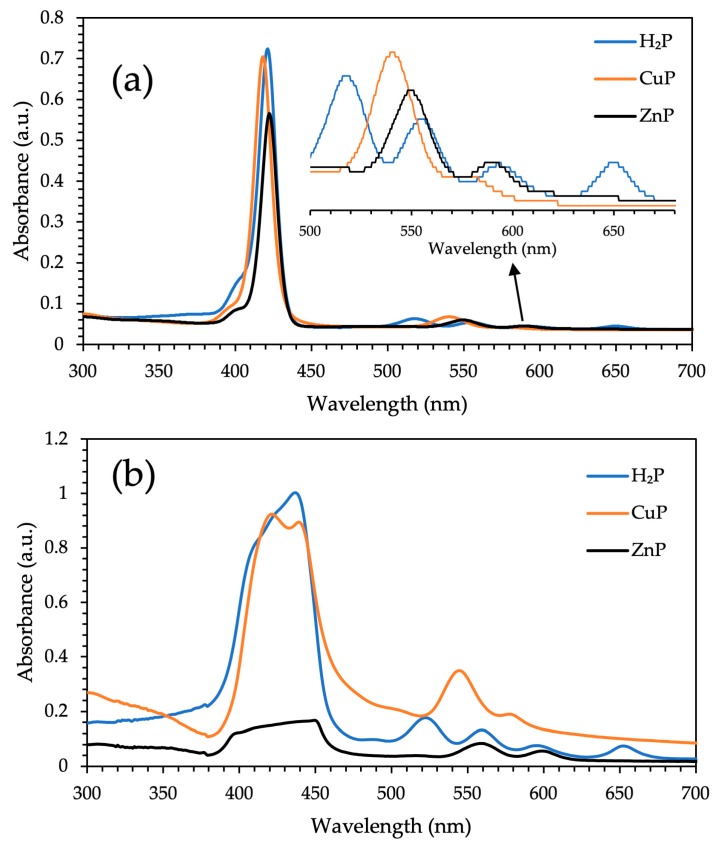
(**a**) UV-Vis absorption spectra of H_2_P, CuP, and ZnP in CH_2_Cl_2_, and in (**b**) solid state.

The UV-visible spectra of the compounds H_2_P, ZnP, and CuP were measured in CH_2_Cl_2_ and thin films, as shown in Figure 1, and the corresponding data are summarized in Table 1. The spectrum exhibits characteristic bands of porphyrins in two distinct regions derived from π → π* transitions of the highly conjugated aromatic porphyrin ring. The most intense band (Soret band) around 400 nm, emerged from transitions of the ground state to the second excited state (S_0_ → S_2_), while bands with molar absorbance magnitude lower (Q bands) between 500–700 nm are assigned to transitions to the first excited state (S_0_ → S_1_) [28,29].

As depicted in Figure 1a, the absorption spectrum of H_2_P shows a strong Soret band at 421 nm, and four shorter Q bands at 515, 556, 592, and 650 nm. After the introduction of the metal atom to the porphyrin core, the number of Q-bands was reduced due to increased symmetry. As expected, ZnP displayed two Q bands at 550 and 590 nm, while CuP exhibits these bands at 541 and 579 nm. In addition, the metalloporphyrins showed blue or redshifts in their Soret bands when compared to free-base due to interactions between the atomic orbitals of metallic center and π-orbitals of the porphyrinic macrocycle [28]. The solid-state absorption spectra of the compounds were obtained from the film, deposited by spin-coating onto glass (Figure 1b). H_2_P and ZnP exhibits a red shift of the Soret band of 16 and 28 nm, respectively, compared to its solution, whereas for the CuP, no shift was observed. The small difference between the film and solution-processed compounds could be due to the formation of aggregates of porphyrins [30], which will be discussed further.

**Figure 2:**
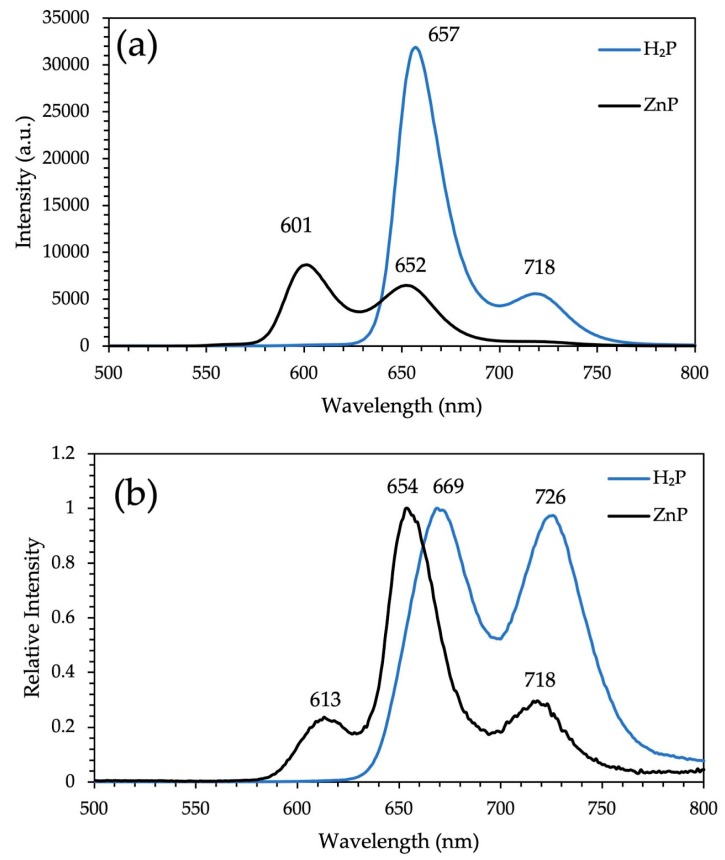
Photoluminescence spectra of H_2_P and ZnP excited at 421 nm in CH_2_Cl_2_ at 10^−6^ M (**a**), and normalized spectra of the solid state of H_2_P and ZnP (**b**).

Figure 2 shows the photoluminescence (PL) spectra of H_2_P, CuP, and ZnP recorded both in solution and in thin film (data in Table 1). In Figure 2a, upon 421 nm photoexcitation, H_2_P showed two bands, a strong peak at 657 nm and a weaker band at 718 nm, which is a typical fluorescence spectrum of porphyrin in the red region. The emission spectrum from ZnP exhibited a maximum band at 601 nm and a smaller band at 652 nm, with a blue-shift in relation to H_2_P due to influence of the metal. Fluorescence quantum yield values for H_2_P and ZnP were similar (Table 1) and agree with the reported literature [29]. CuP did not exhibit fluorescence under the same conditions.

Figure 2b shows the emission spectra of H_2_P and ZnP in film. Comparing with the spectra of the Figure 2a, differences are observed in the spectral profile as well as a red-shift in about 12 nm. This was due to the formation of aggregates porphyrins in the solid state. It has been reported that the π-conjugation of porphyrins causes intermolecular interactions, which depending on the morphology of the aggregation, can be classified into type H or J. In the H-type aggregates, molecules are aligned parallel to each other (face-to-face), and the emission bands are blue-shifted and generally lead to the fluorescence quenching. In J-type aggregates, the molecules are arranged in the edge-to-edge direction, inducing a red-shift in absorption and emission bands, and remain optically allowed [31,32,33].

**Figure 3:**
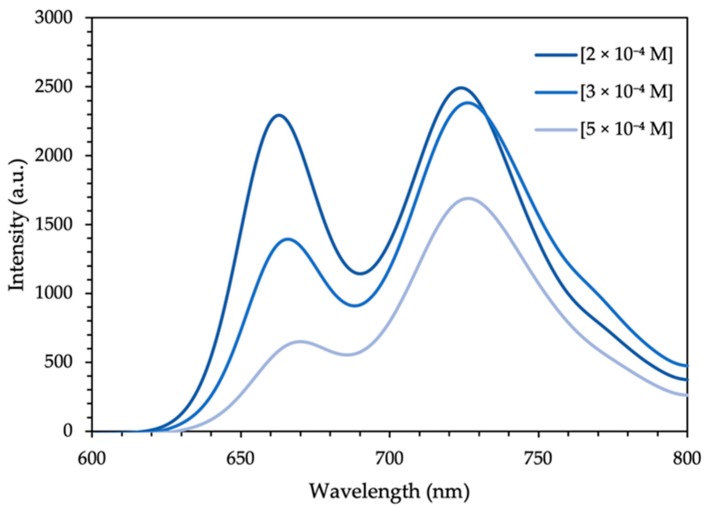
Emission spectra of H_2_P in different concentrations.

To confirm the formation of aggregates, a fluorescence emission analysis was performed varying the concentration in solution, as shown in Figure 3. It was observed that the modification in concentration altered the spectral profile without causing fluorescence quenching, indicating the formation of J-aggregates [32,34,35]. Besides that, we verified that the profile obtained in the film (Figure 2b) had the same characteristic as the one obtained from a 2 × 10^−4^ M solution. In this material, the introduction of a bulky substituents hindered the π–π stacking and induced the formation of J-aggregate favoring the emission in the aggregated solid state [32,33].

As mentioned above, the CuP did not show any fluorescence in solution. It is known that copper is a paramagnetic atom (with an unpaired electron in the d orbital) that causes fluorescence quenching due to the mixing of spin multiplicity from the paramagnetic metallic center and the porphyrin ring itself, increasing the intersystem crossing process [36,37,38].

**Figure 4:**
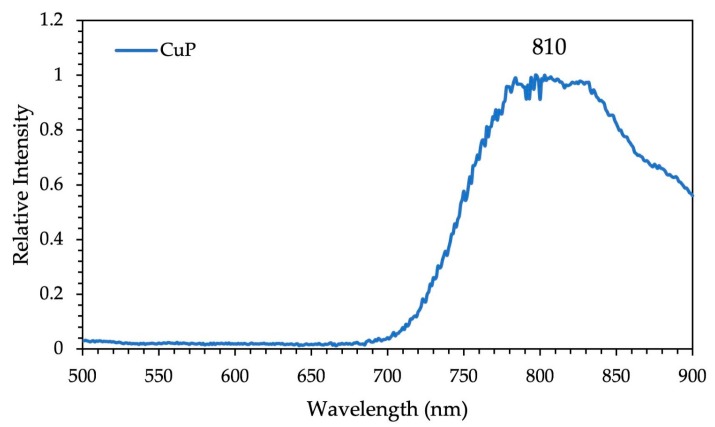
Photoluminescence of CuP in film (λ_exc_ = 435 nm).

However, in the solid state (aggregate form), CuP exhibited luminescence (Figure 4). In the aggregated state, the CuP complex came from process ‘‘aggregation-induced emission’’ (AIE) due to the restriction effect of intramolecular rotations (RIR) caused by the J-aggregates that block the non-radiative pathway allowing radioactive decay in the NIR region [31,33,39].

### 3.2. Electrochemical Properties

Cyclic voltammetry (CV) was used to evaluate the redox properties of H_2_P, CuP, and ZnP in a CH_2_Cl_2_ solution [1 mM] containing 50 mM of TBAPF_6_ as a supporting electrolyte under a nitrogen atmosphere. The highest occupied molecular orbital (HOMO) and lowest unoccupied molecular orbital (LUMO) energy levels were determined from the oxidation and reduction potentials, respectively.

The CV curves were calibrated using the ferrocenium/ferrocene (Fc^+^/Fc) redox couple as an external standard. The HOMO and LUMO energy levels were obtained through Equations (1) and (2) [40], where *e* is the electron charge, *φ* is the correction factor between the ferrocene value in the literature with the observed during the measures, $E_{onset}^{oxi}$ (V) and $E_{onset}^{red}$ (V) are, respectively, the onset potentials for oxidation and reduction relative to the standard hydrogen electrode (SHE).

(1) $$E_{HOMO}\left(eV\right)=e.E_{onset}^{oxi}\left(V\right)+4.4\left(eV\right)-e.\varphi \left(V\right)$$

(2) $$E_{LUMO}\left(eV\right)=e.E_{onset}^{red}\left(V\right)+4.4\left(eV\right)-e.\varphi \left(V\right)$$

All data are compiled in Table 2. The free base H_2_P exhibited in the cathodic sense one reversible reduction wave with E½ = −1.23 V, which corresponds to the reduction of the porphyrin ring. By changing the sweep direction to the most positive range, two reversible oxidation waves in E½ = 0.93 V and E½ = 1.27 V were observed and can be attributed to the first and second monoelectronics oxidation of the porphyrin ring. For the ZnP and CuP complexes, there were small changes of the oxidation and reduction potentials to more negative values (see Supporting Information).

According to Stute and coworkers [41], the E½ values depended on the number and nature of donor atoms in the porphyrin core. However, the influence of Cu(II) and Zn(II) on the reduction potentials was not as distinct as that of the ligand. All energy gap values, known as electrochemical gaps, were close to the usual value for porphyrins and metalloporphyrins [41,42], and to the theoretical value of 2.18 eV given by Gouterman et al. [43].

### 3.3. Fabrication of the OLEDs Ddevices

The structure of devices consisted in a tri-layer architecture constructed onto ITO (indium tin oxide)-coated glass substrates with a sheet resistance of 15 Ω square^−1^ (anode). The poly(3,4-ethylenedioxythiophene)-poly(styrenesulfonate) (PEDOT:PSS), used as a hole transporting layer, was deposited via spin coating onto the ITO. The emitting layer (H_2_P, ZnP, and CuP) was deposited via spin-coating using tetrahydrofuran (THF) as a solvent. The electron transporting layer, 1,3,5-tris(1-phenyl-1Hbenzimidazol-2-yl)benzene (TPBi), as well as LiF and Al films, were successively deposited via thermal deposition.

**Figure 5:**
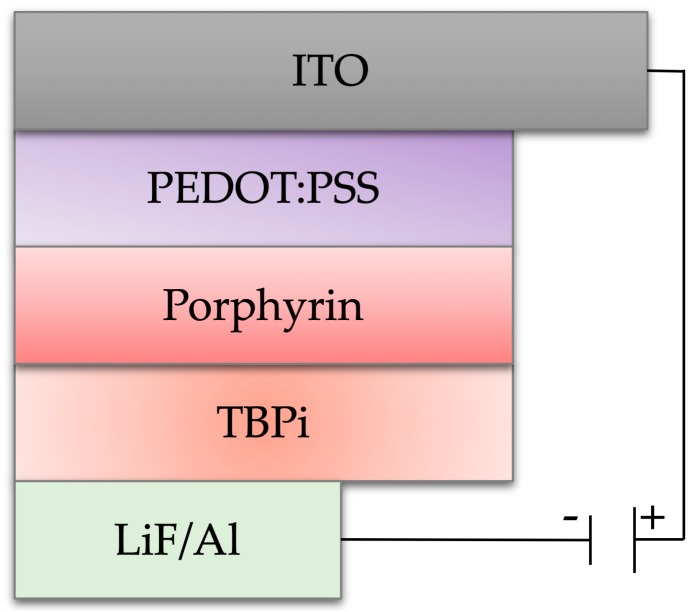
Schematic structure of the OLEDs.

The resulting devices, with the respective layers thicknesses (nm) (Figure 5), were:

OLED-1 ITO/PEDOT:PSS(40)/H_2_P(30)/TPBi(25)/LiF(0.1)/Al(100)

OLED-2 ITO/PEDOT:PSS(40)/ZnP(30)/TPBi(25)/LiF(0.1)/Al(100)

OLED-3 ITO/PEDOT:PSS(40)/CuP(30)/TPBi(25)/LiF(0.1)/Al(100)

### 3.4. Electroluminescence (EL)

**Figure 6:**
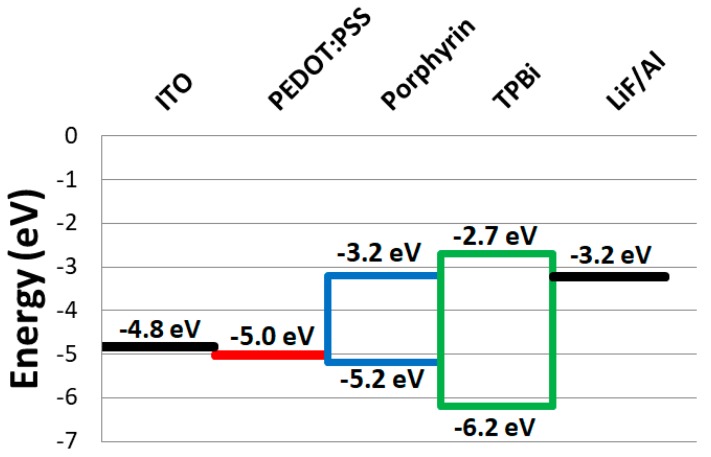
Energy level diagram of the OLEDs.

The energy level diagram shown in Figure 6 was drawn based on the values of the HOMO and LUMO energy levels determined from the cyclic voltammetry. The HOMO levels for porphyrins were around 5.2 eV, indicating an energy barrier of 0.2 eV for hole injection when compared with PEDOT:PSS. On the other hand, an electron barrier of about 0.5 eV was present between the LiF/Al electrode and the LUMO TPBi levels, indicating that for all OLEDs fabricated in this study, the holes were more efficiently injected than electrons, and in the interface porphyrins/TPBi, the holes were accumulated by an energy barrier of 1.0 eV, increasing the probability of recombination in the porphyrins.

**Figure 7:**
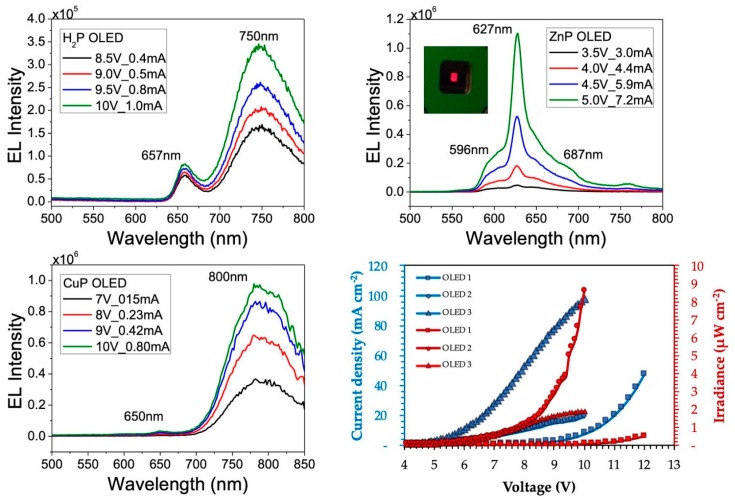
Electroluminescence spectra and the irradiance and current density curves for the devices OLED-1 (H_2_P), OLED-2 (ZnP), and OLED-3 (CuP).

In Figure 7 are shown the EL spectra of OLEDs 1, 2, and 3 as a function of applied voltage and current (top), and the irradiance versus current density curves for the devices (bottom). For OLED-1 (Figure 7a), the shape (halfwidth) of the emission band was independent of the driving voltage values, indicating that the charges carriers generated excitons within the H_2_P layer. The EL spectrum of the OLED-1 had two bands, one of lower intensity at 650 nm and another more intense one at 750 nm. By varying the applied voltage from 8 V to 10 V, the peak at 657 nm showed a small variation of intensity, while the peak at 750 nm increased proportionally to the voltage. The EL emission spectrum of OLED-1 matched the photoluminescence (PL) spectrum of Figure 4 in the formation of J-aggregates characterized by the increase in the concentration involved in the film. A possible explanation for this emission is the formation of exciplexes at the porphyrin/TPBi interface in a non-equilibrium regime such as the application of an external electric field. In this case, the energy levels were depleted, deviating from the rigid energy levels alignment (showed in Figure 6). In fact, this energy-level diagram should be interpreted as an initial guide for engineering the devices’ heterojunctions. At the interfaces, several interactions can lead to changes in the electronic structure resulting in optical absorption/emissions that deviate from what one should expect by looking at the rigid diagram. The same feature was previously reported in the literature [44,45]. The threshold voltage (V_on_) of the OLED-1 was 8 V and the maximum irradiance observed was 0.5 μW cm^−2^ at a current density (J) of 47 mA cm^−2^ at 12 V (Figure 7d).

The EL spectrum of OLED-2 is shown in Figure 7b. The EL emission spectrum of OLED-2 matched the PL spectrum of ZnP as a thin film and was slightly blue-shifted, with an intense peak observed at 627 nm and two smaller peaks at 597 and 687 nm. The threshold voltage (V_on_) was 4 V, and the maximum irradiance was 8.5 μW cm^−2^ with a current density (J) of 19 mA cm^−2^ at 10 V (Figure 7d). The EL spectrum of OLED-3 had a band in the near-IR at 800 nm (Figure 7c) with a threshold voltage (V_on_) of 4V and a maximum irradiance of 2 μW cm^−2^ with a current density (J) of 97 mA cm^−2^ at 10 V (Figure 7d). These results indicate the superior performance of the ZnP porphyrin as an OLED emitting layer compared to H_2_P and CuP.

The interaction between the atomic orbitals of Zn and π-orbitals of the porphyrinic macrocycle, allowed for OLED-2 to increase the irradiance (almost 9 times for H2P and 4 times for CuP), as well as decreasing the current of the device and increasing its lifetime.

Several studies use porphyrin as a dopant, in which they increased the number of layers, and the device performance depended on the effective overlap of energy transfer from the electron carrier (host) to the dopant (guest). Therefore, it is important to highlight that in this study, the porphyrins were not used as dopants, and that they showed an electroluminescence in the red and a threshold voltage, which is similar to that reported in other works involving porphyrins as an emitter layer [8,9,14,15,46,47].

## 4. Conclusions

We reported the application of a CNSL-derived porphyrin and metaloporphyrins, as an emitting layer in OLEDs, proposing a more sustainable way to produce these devices. All materials were used as emitting layers in OLEDs, and their electroluminescence properties were studied. The ZnP-based device presented emission at 627 nm, while the CuP emitted in the near infrared at 800 nm. CuP complex was a promising material for the design of efficient and stable OLEDs in the infrared. ZnP had superior stability and irradiance that were 9-fold and 4-fold higher than the OLEDs with H_2_P and CuP, respectively. These results are relevant because they represent an interesting alternative in the production of optoelectronic materials using a more sustainable and efficient route, since they are derived from a by-product of the cashew nut agroindustry. Additionally, the replacement of heavy metals by more abundant metals (zinc and copper, for example) and a simpler device can stimulate the development of low cost eco-friendly materials. Current technological advances should seek the sustainability on the designing of new products, in particular, materials for optoelectronic applications.

## Figures and Tables

**Table 1:** Absorption and emission data for compounds H_2_P, CuP, and ZnP in CH_2_Cl_2_ at 298 K.

	UV-vis λ_max_ (nm) Soret; Q (log *ε*_max_/ M^−1^ cm^−1^)		PL λ_max_ (nm)		
Compound	In Solution ^a^	In Film ^b^	In Solution	In Film	Φ_F_
H_2_P	421 (5.6); 515 (4.3); 556 (4.2); 592 (4.1); 650 (4.1)	437; 522; 559; 596; 652	657; 718	669; 726	0.17
CuP	418 (5.5); 541 (4.2); 579 (4.0)	421; 545	____	810	____
ZnP	423 (5.6); 550 (4.3); 590 (4.1)	451; 560	601; 652	613; 654; 718	0.13

^a^ Measured in CH_2_Cl_2_ (2 × 10^−6^ M) solution. ^b^ Measured from thin films on glass. ^c^ Fluorescence quantum yield measured using tetrapyridylporphyrin (ΦF = 0.069) as standard.

**Table 2:** Electrochemical data of H_2_P, ZnP, and CuP at 25 °C in CH_2_Cl_2_.

Compound	E_HOMO_ (eV)	E_LUMO_ (eV)	GAP (eV)
H_2_P	−5.20	−3.20	2.00
ZnP	−5.10	−3.00	2.10
CuP	−5.20	−3.00	2.20

## Supplementary Materials

The following are available online at https://www.mdpi.com/1996-1944/12/7/1063/s1, Figure S1. ^1^H NMR (CDCl_3_, 500 MHz) spectrum of free base porphyrin (H_2_P).; Figure S2. ^1^H NMR (CDCl_3_, 500 MHz) spectrum of zinc porphyrin (ZnP).; Figure S3. ^1^H NMR (CDCl_3_, 500 MHz) spectrum of copper porphyrin (CuP).; Figure S4. ^13^C NMR spectrum of H_2_P (500 MHz, CDCl_3_).; Figure S5. ^13^C NMR spectrum of ZnP (500 MHz, CDCl_3_).; Figure S6. ^13^C NMR spectrum of CuP (500 MHz, CDCl_3_).; Figure S7. MS (MALDI-TOF) of H_2_P m/z: calcd for 2000.9224 u; found [M+H^+^] 2000.0000 u.; Figure S8. MS (MALDI-TOF) of ZnP *m/z*: calcd for 2064.2966 u; found [M+H^+^] 2064.0000 u.; Figure S9. MS (MALDI-TOF) of CuP m/z: calcd for 2062.4526 u; found [M+H^+^] 2062.0000 u.; Figure S10. X-ray powder diffraction of CuP, H_2_P and ZnP.; Figure S11. Cyclic voltammogram of H_2_P in CH_2_Cl_2_, 50 mM of TBAPF_6_. Scan rate = 50 mVs^−1^.; Figure S12. Cyclic voltammogram of ZnP in CH_2_Cl_2_, 50 mM of TBAPF_6_. Scan rate = 50 mV/s.; Figure S13. Cyclic voltammogram of CuP in CH_2_Cl_2_, 50 mM of TBAPF_6_. Scan rate = 50 mVs^−1^.; Figure S14. Fluorescence excitation spectrum of H_2_P monitored at 657 nm.; Figure S15. Fluorescence excitation spectrum of ZnP monitored at 601 nm.

## References

[1] Giovanella U., Pasini M., Botta C., Bergamini G., Silvi S. (2016). Organic Light-Emitting Diodes (OLEDs): working principles and device technology. Applied Photochemistry.

[2] Graham K.R., Yang Y., Sommer J.R., Shelton A.H., Schanze K.S., Xue J., Reynolds J.R. (2011). Extended Conjugation Platinum(II) Porphyrins for use in Near-Infrared Emitting Organic Light Emitting Diodes. Chem. Mater..

[3] Vasilopoulou M., Georgiadou D.G., Soultati A., Douvas A.M., Papadimitropoulos G., Davazoglou D., Pistolis G., Stathopoulos N.A., Kamalakis T., Alexandropoulos D. (2015). Solution processed multi-color organic light emitting diodes for application in telecommunications. Microelectron Eng..

[4] Wang J., Zhang F., Zhang J., Tang W., Tang A., Peng H., Xu Z., Teng F., Wang Y. (2013). Key issues and recent progress of high efficient organic light-emitting diodes. J. Photochem. Photobiol. C.

[5] Volz D., Wallesch M., Fléchon C., Danz M., Verma A., Navarro J.M., Zink D.M., Bräse S., Baumann T. (2015). From iridium and platinum to copper and carbon: new avenues for more sustainability in organic light-emitting diodes. Green Chem..

[6] Quirino W.G., Teixeira K.C., Legnani C., Calil V.L., Messer B., Vilela Neto O.P., Pacheco M.A.C., Cremona M. (2009). Improved multilayer OLED architecture using evolutionary genetic algorithm. Thin Solid Films.

[7] Bagatin I.A., Legnani C., Cremona M. (2009). Investigation on Al(III) and Zn(II) complexes containing a calix [4] arene bearing two 8-oxyquinoline pendant arms used as emitting materials for OLEDs. Mater. Sci. Eng. C.

[8] Roth C.O.P., Drouet S., Merhi A., Williams J.A.G., Gildea L.F., Pearson C., Petty M.C. (2013). Synthesis of platinum complexes of fluorenyl-substituted porphyrins used as phosphorescent dyes for solution-processed organic light-emitting devices. Tetrahedron.

[9] Janghouri M., Adineh M. (2017). Color optimization of red organic light emitting diodes (OLEDs) through dihydroxyphenyl-substituted zinc porphyrins emitters. J. Photochem. Photobiol. A.

[10] Barrientos H., Arias E., Moggio I., Romero J., Rodriguez O., Giorgetti E., Del Rosso T. (2004). Dodecanoxy-phenylethynylene oligomers for light emitting diodes. Synth. Met..

[11] Shan T., Gao Z., Tang X., He X., Gao Y., Li J., Sun X., Liu Y., Liu H., Yang B. (2017). Highly efficient and stable pure blue nondoped organic light-emitting diodes at high luminance based on phenanthroimidazole-pyrene derivative enabled by triplet-triplet annihilation. Dyes Pigm..

[12] Gudeika D., Volyniuk D., Mimaite V., Lytvyn R., Butkute R., Bezvikonnyi O., Buika G., Grazulevicius J.V. (2017). Carbazolyl-substituted quinazolinones as high-triplet-energy materials for phosphorescent organic light emitting diodes. Dyes Pigm..

[13] Wang P., Fan S., Liang J., Ying L., You J., Wang S., Li X. (2017). Carbazole-diphenylimidazole based bipolar material and its application in blue, green and red single layer OLEDs by solution processing. Dyes Pigm..

[14] Jafari M.R., Bahrami B. (2015). Emission properties of porphyrin compounds in new polymeric PS: CBP host. Appl. Phys. A.

[15] Shahroosvand H., Zakavi S., Sousaraei A., Mohajeranic E., Mahmoudic M. (2015). Unusual near-white electroluminescence of light emitting diodes based on saddle-shaped porphyrins. Dalton Trans..

[16] Lomonaco D., Mele G., Mazzetto S.E., Anilkumar P. (2017). Cashew Nutshell Liquid (CNSL): from an agro-industrial waste to a sustainable alternative to petrochemical resources. Cashew Nut Shell Liquid: A Goldfield for Functional Materials.

[17] Mele G., Lomonaco D., Mazzetto S.E., Anilkumar P. (2017). Cardanol-based heterocycles: synthesis and applications. Cashew Nut Shell Liquid.

[18] Ribeiro V.G.P., Marcelo A.M.P., Silva K.T., Silva F.L.F., Mota J.P.F., Nascimento J.P.C., Sombra A.S.B., Clemente C.S., Mele G., Carbone L. (2017). New ZnO@Cardanol Porphyrin Composite Nanomaterials with Enhanced Photocatalytic Capability under Solar Light Irradiation. Materials.

[19] Vasapollo G., Mele G., del Sole R., Pio I., Li J., Mazzetto S.E. (2011). Use of Novel Cardanol-Porphyrin Hybrids and Their TiO_2_-Based Composites for the Photodegradation of 4-Nitrophenol in Water. Molecules.

[20] Attanasi O.A., Berretta S., Fiani C., Filippone P., Mele G., Saladino R. (2006). Synthesis and reactions of nitro derivatives of hydrogenated cardanol. Tetrahedron.

[21] Mota J.P.F., Júnior A.E.C., Ribeiro V.G.P., Sampaio S.G., Lima N.M.A., Silva F.L.F., Clemente C.S., Mele G., Lomonaco D., Mazzetto S.E. (2016). Synthesis, Characterization and Dielectric Properties of New 5-(4-Hydroxyphenyl)-10,15,20-tri-4-[2-(3-pentadecylphenoxy)ethoxy] phenyl porphyrin and Their Ni, Co and Cu Complexes. J. Braz. Chem. Soc..

[22] Voirin C., Caillol S., Sadavarte N.V., Tawade B.V., Boutevin B., Wadgaonkar P.P. (2014). Functionalization of cardanol: towards biobasedpolymers and additives. Polym. Chem..

[23] Clemente C.S., Ribeiro V.G.P., Sousa J.E.A., Maia F.J.N., Barreto A.C.H., Andrade N.F., Denardin J.C., Mele G., Carbone L., Mazzetto S.E. (2013). Porphyrin synthesized from cashew nut shell liquid as part of a novel superparamagnetic fluorescence nanosystem. J Nanopart Res..

[24] Deyab M.A., Mele G., Al-Sabagh A.M., Bloise E., Lomonaco D., Mazzetto S.E., Clemente C.D.S. (2017). Synthesis and characteristics of alkyd resin/M-Porphyrins nanocomposite for corrosion protection application. Prog. Org. Coat..

[25] Sandrino B., Clemente C.D.S., Oliveira T.M.B.F., Ribeiro F.W.P., Pavinatto F.J., Mazzetto S.E., Neto P.L., Correia A.N., Pessoa C.A., Wohnrath K. (2013). Amphiphilic porphyrin-cardanol derivatives in Langmuir and Langmuir–Blodgett films applied for sensing. Colloids Surf. A.

[26] Bloise E., Carbone L., Colafemmina G., D’Accolti L., Mazzetto S.E., Vasapollo G., Mele G. (2012). First Example of a Lipophilic Porphyrin-Cardanol Hybrid Embedded in a Cardanol-Based Micellar Nanodispersion. Molecules.

[27] Attanasi O.A., del Sole R., Filipponea P., Mazzetto S.E., Mele G., Vasapollo G. (2004). Synthesis of novel lipophilic porphyrin-cardanol derivatives. J. Porphyrins Phthalocyanines.

[28] Valicsek Z., Horváth O. (2013). Application of the electronic spectra of porphyrins for analytical purposes: The effects of metal ions and structural distortions. Microchem. J..

[29] Xu Z., Mei Q., Hua Q., Tian R., Weng J., Shi Y., Huang W. (2015). Synthesis, characterization, energy transfer and photophysical properties of ethynyl bridge linked porphyrin–naphthalimide pentamer and its metal complexes. J. Mol. Struct..

[30] Kumar P.R., Mothi E.M., Ramesh M., Kathiravan A. (2018). Zn Porphyrin propped with hydantoin anchor: synthesis, photophysics and electron injection/recombination dynamics. Phys. Chem. Chem. Phys..

[31] Verma S., Ghosh H.N. (2012). Exciton Energy and Charge Transfer in Porphyrin Aggregate/Semiconductor (TiO_2_) Composites. J. Phys. Chem. Lett..

[32] Zhang G., Chen Q., Zhang Y., Kong L., Tao X., Lu H., Tian Y., Yang J. (2014). Bulky group functionalized porphyrin and its Zn (II) complex with high emission in aggregation. Inorg. Chem. Commun..

[33] Hong Y., Lam J.W.Y., Tang B.Z. (2011). Aggregation-induced emission. Chem. Soc. Rev..

[34] Jana A., McKenzie L., Wragg A.B., Ishida M., Hill J.P., Weinstein J.A., Baggaley E., Ward M.D. (2016). Porphyrin/platinum(II) C^N^N acetylide complexes: synthesis, photophysical properties, and singlet oxygen generation. Chem. Eur. J..

[35] Xu X.D., Zhang J., Chen L.J., Guo R., Wang D.X., Yang H.B. (2012). Design and synthesis of branched platinum–acetylide complexes possessing a porphyrin core and their self-assembly behaviour. Chem. Commun..

[36] Fushimi Y., Koinuma M., Yasuda Y., Nomura K., Asano M.S. (2017). Effects of end-groups on photophysical properties of poly(9,9-di-*n*-octylfluorene-2,7-vinylene)s linked with metalloporphyrins: synthesis and time-resolved fluorescence spectroscopy. Macromolecules.

[37] Szintay G., Horva’th A. (2001). Five-coordinate complex formation and luminescence quenching study of copper(II) porphyrins. Inorg. Chim. Acta.

[38] Milot R.L., Moore G.F., Crabtree R.H., Brudvig G.W., Schmuttenmaer C.A. (2013). Electron injection dynamics from photoexcited porphyrin dyes into SnO_2_ and TiO_2_ nanoparticles. J. Phys. Chem. C.

[39] Verma S., Ghosh A., Das A., Ghosh H.N. (2010). Ultrafast exciton dynamics of J- and H-Aggregates of the porphyrin-catechol in aqueous solution. J. Phys. Chem. B.

[40] Ranjith K., Swathi S.K., Kumar P., Ramamurthy P.C. (2012). Dithienylcyclopentadienone derivative-co-benzothiadiazole: An alternating copolymer for organic photovoltaics. Sol. Energy Mater. Sol. Cells.

[41] Stute S., Götzke L., Meyer D., Merroun M.L., Rapta P., Kataeva O., Seichter W., Gloe K., Dunsch L., Gloe K. (2013). Molecular Structure, UV/Vis Spectra, and Cyclic Voltammograms of Mn(II), Co(II), and Zn(II) 5,10,15,20-Tetraphenyl-21-oxaporphyrins. Inorg. Chem..

[42] Nasri S., Zahou I., Turowska-Tyrk I., Roisnel T., Loiseau F., Saint-Amant E., Nasri H. (2016). Synthesis, electronic spectroscopy, cyclic voltammetry, photophysics, electrical properties and X-ray molecular structures of *meso* -{Tetrakis[4-(benzoyloxy)phenyl]porphyrinato}zinc(II) complexes with aza ligands. Eur. J. Inorg. Chem..

[43] Zerner M., Gouterman M. (1966). Porphyrins - IV. Extended Hückel calculations on transition metal complexes. Theor. Chim. Acta.

[44] Li J., Nomura H., Miyazaki H., Adachi C. (2014). Highly efficient exciplex organic light-emitting diodes incorporating a heptazine derivative as an electron acceptor. Chem. Commun..

[45] Matsumoto N., Nishiyama M., Adachi C. (2008). Exciplex formations between tris(8-hydoxyquinolate)aluminum and hole transport materials and their photoluminescence and electroluminescence characteristics. J. Phys. Chem. C.

[46] Zhu L.J., Wang J., Reng T.G., Li C.Y., Guo D.C., Guo C.C. (2010). Effect of substituent groups of porphyrins on the electroluminescent properties of porphyrin-doped OLED devices. J. Phys. Org. Chem..

[47] Andreasson M.H., Martensson J., Andersson T.G. (2008). Porphyrin doping of Alq_3_ for electroluminescence. Curr. Appl Phys..

