# Supplementary material for: Light-Emitting Porphyrin Derivative Obtained from a Subproduct of the Cashew Nut Shell Liquid: A Promising Material for OLED Applications
Source: Materials (Basel). 2019 Apr 1;12(7):1063. doi: 10.3390/ma12071063 (PMC6479845; doi:10.3390/ma12071063)
Supplement: Supplementary file 1 [file materials-12-01063-s001.pdf]

Supporting Information

Light-emitting porphyrin-derivative obtained from a subproduct of the cashew nut shell liquid: a promising material for OLED applications

Nayane Maria de Amorim Lima[1], Harold José Camargo Avila[2,‡], Cleber Fabiano do Nascimento Marchiori[3], Samuel Gondim Sampaio[1], João Paulo Ferreira Mota[1], Viviane Gomes Pereira Ribeiro[4], Claudenilson da Silva Clemente[1], Giuseppe Mele[5], Marco Cremona[2] and Selma Elaine Mazzetto[1]

1 Laboratory of Products and Process Technology (LPT), Organic and Inorganic Chemistry Department, Federal University of Ceara (UFC), Campus do Pici, 60440-900, Fortaleza-CE, Brazil.

2 Molecular Optoelectronic Laboratory (LOEM), Physics Department, Pontifical Catholic University of Rio de Janeiro (PUC-Rio), 22451-900, Rio de Janeiro-RJ, Brazil.

3 Materials Theory Division, Department of Physics and Astronomy, Uppsala University, Uppsala, Sweden.

4 Institute of Exact and Natural Sciences - ICEN, University of International Integration of Afro-Brazilian Lusophony, 62790-000, Redenção-CE, Brazil.

5 Department of Innovation Engineering, University of Salento, Via Arnesano, 73100, Lecce, Italy.

‡ Permanent address: Department of Physics, University of Atlantic, Puerto Colombia, Atlántico, Colombia

* Correspondence: cremona@fis.puc-rio.br; Tel.: +55-21-35271268

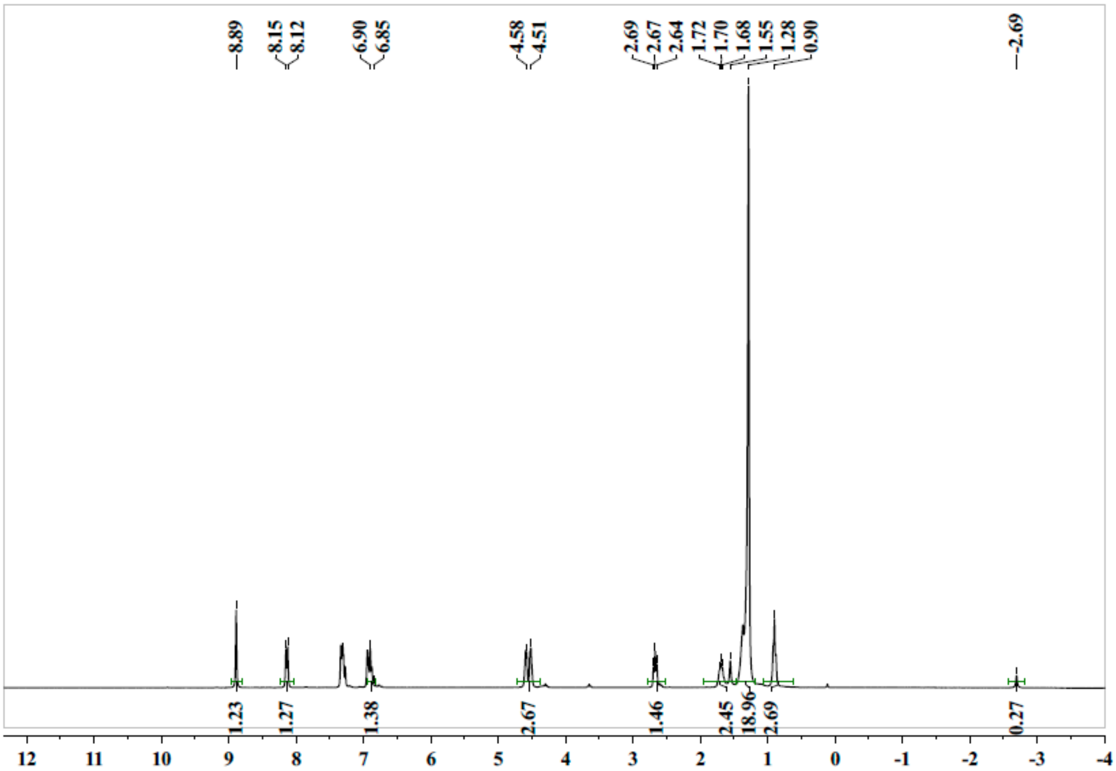

Figure S1. ^{1}H NMR ($CDCl_3$, 500 MHz) spectrum of free base porphyrin (H_2P).

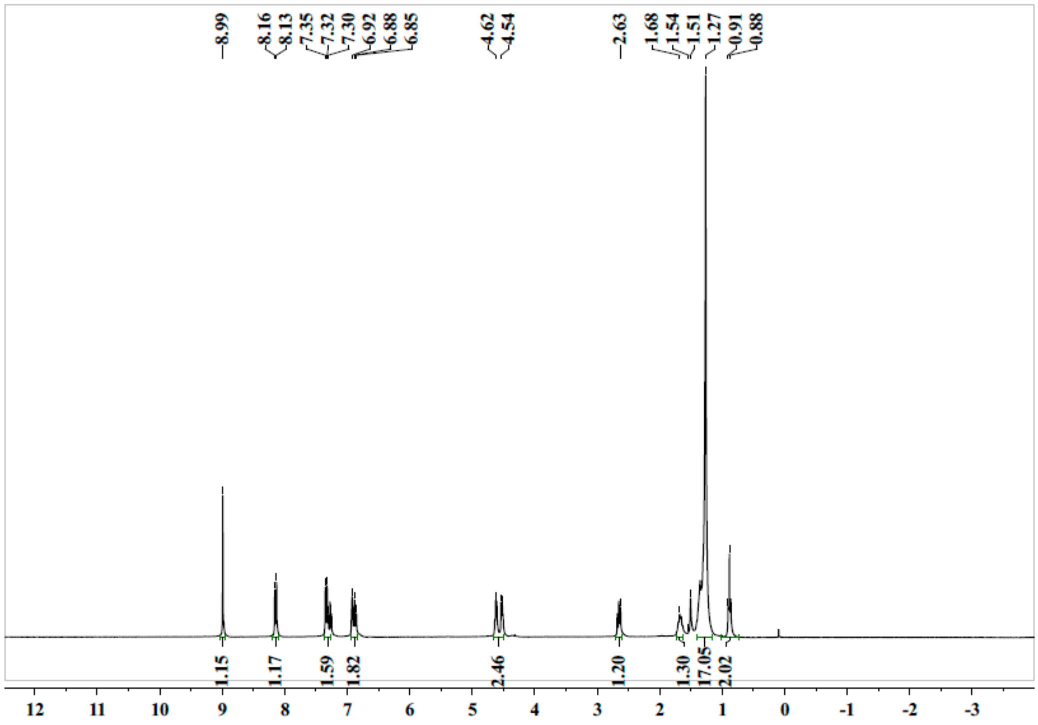

Figure S2. ^{1}H NMR ($CDCl_3$, 500 MHz) spectrum of zinc porphyrin (ZnP).

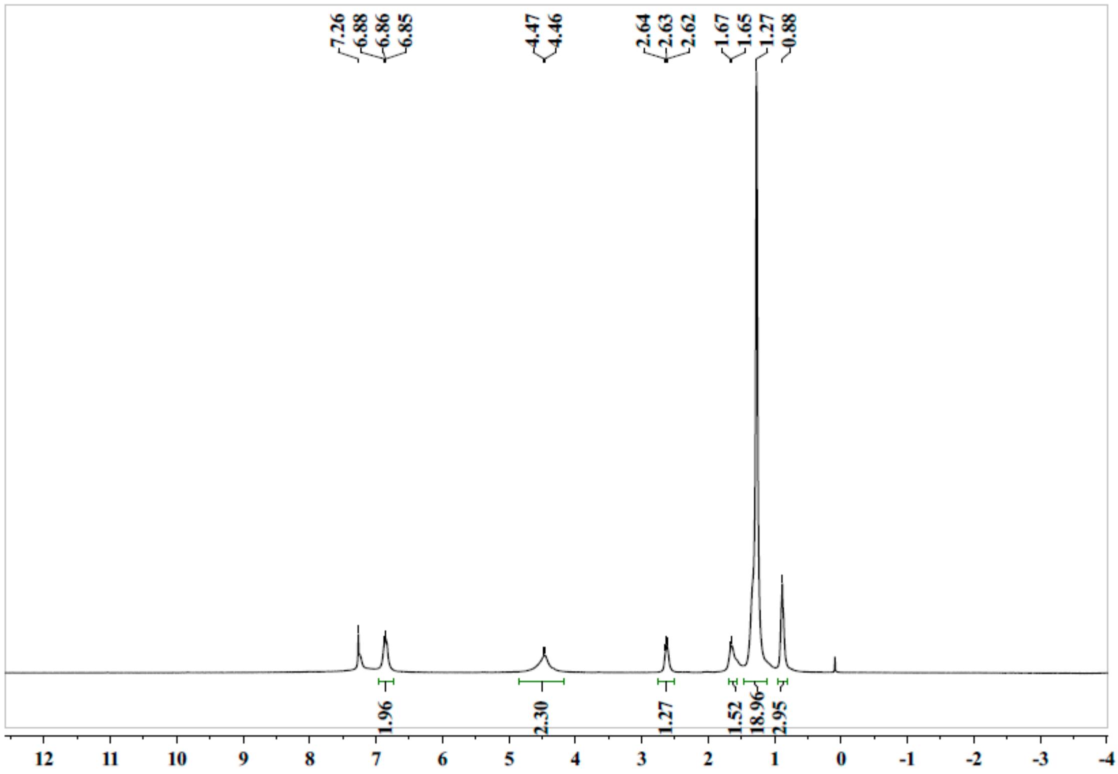

Figure S3. ^{1}H NMR ($CDCl_3$, 500 MHz) spectrum of copper porphyrin (CuP).

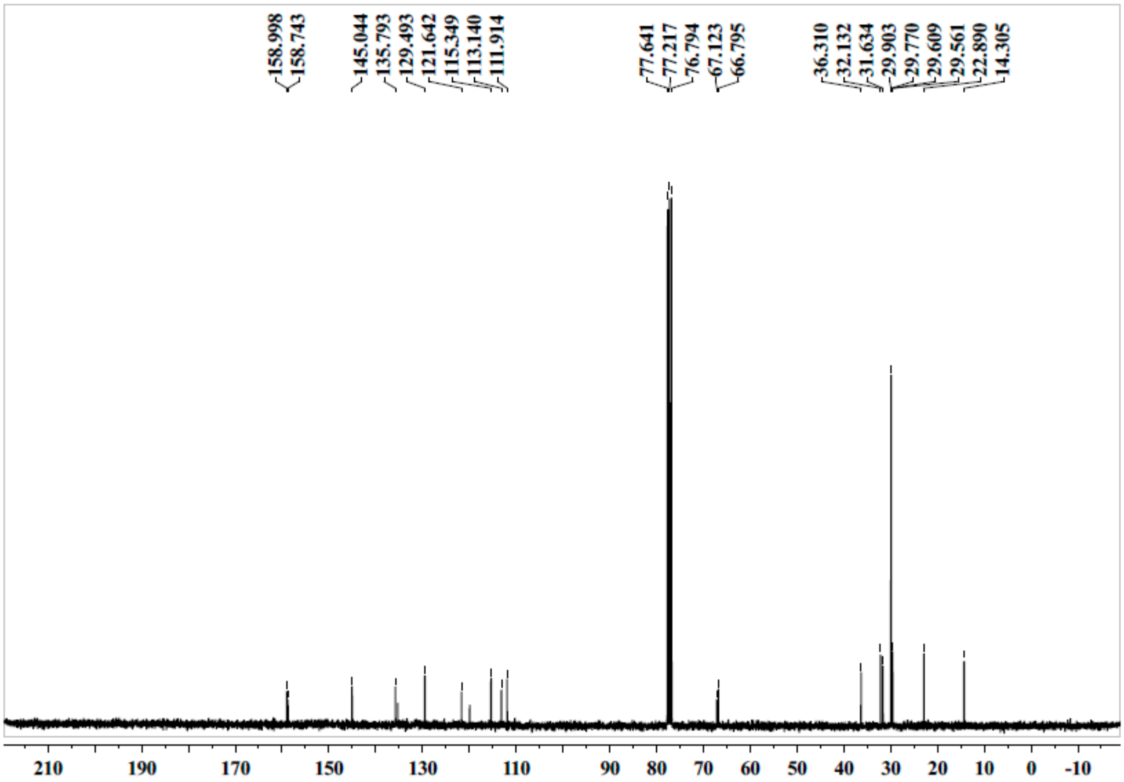

Figure S4. ^{13}C NMR spectrum of H_2P (500 MHz, $CDCl_3$).

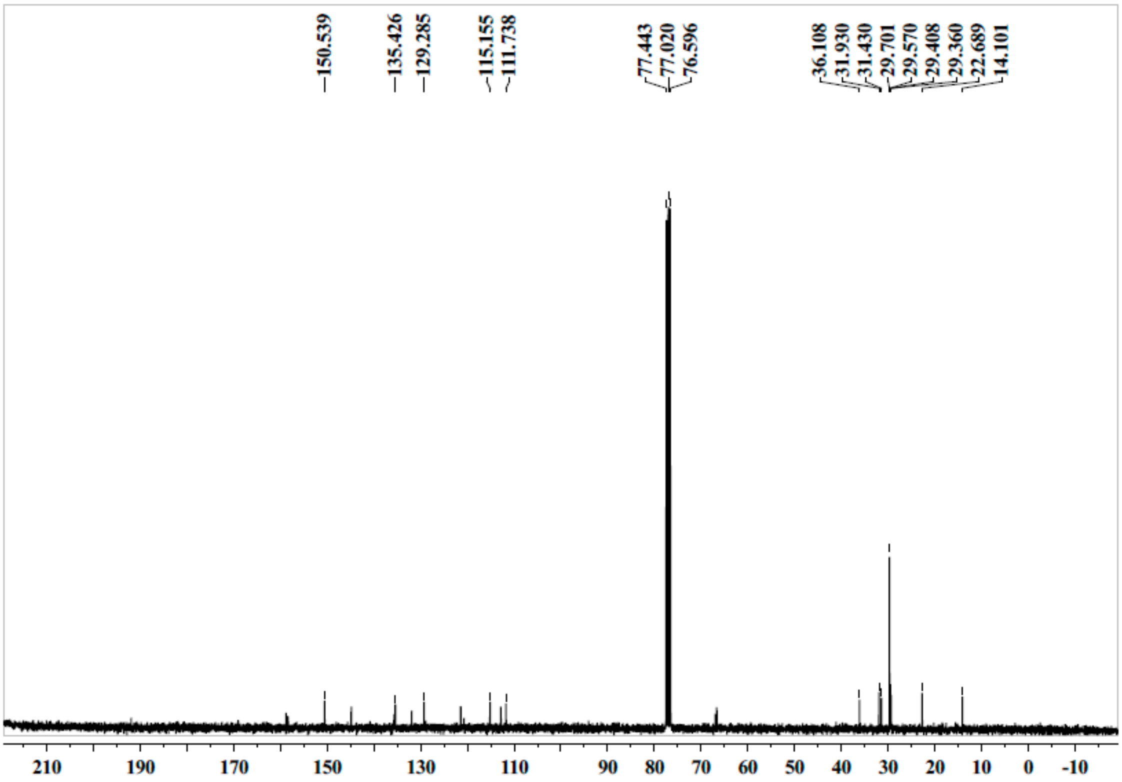

Figure S5. ^{13}C NMR spectrum of ZnP (500 MHz, $CDCl_3$).

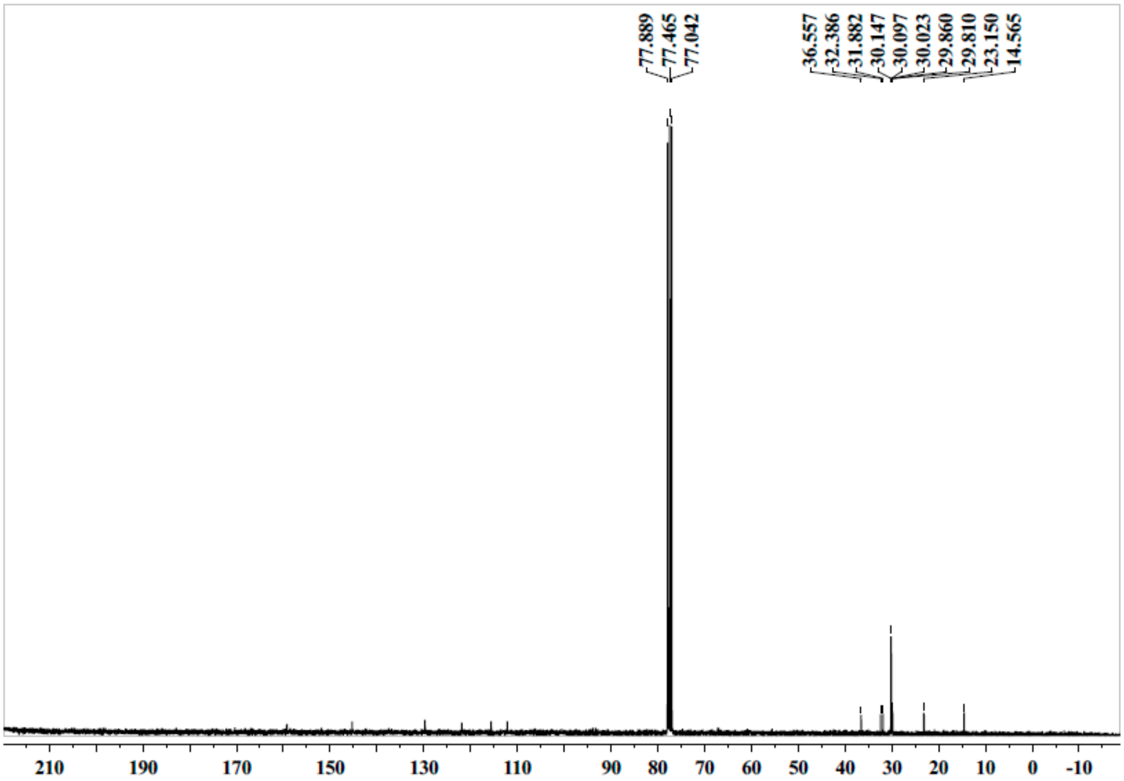

Figure S6. ^{13}C NMR spectrum of CuP (500 MHz, $CDCl_3$).

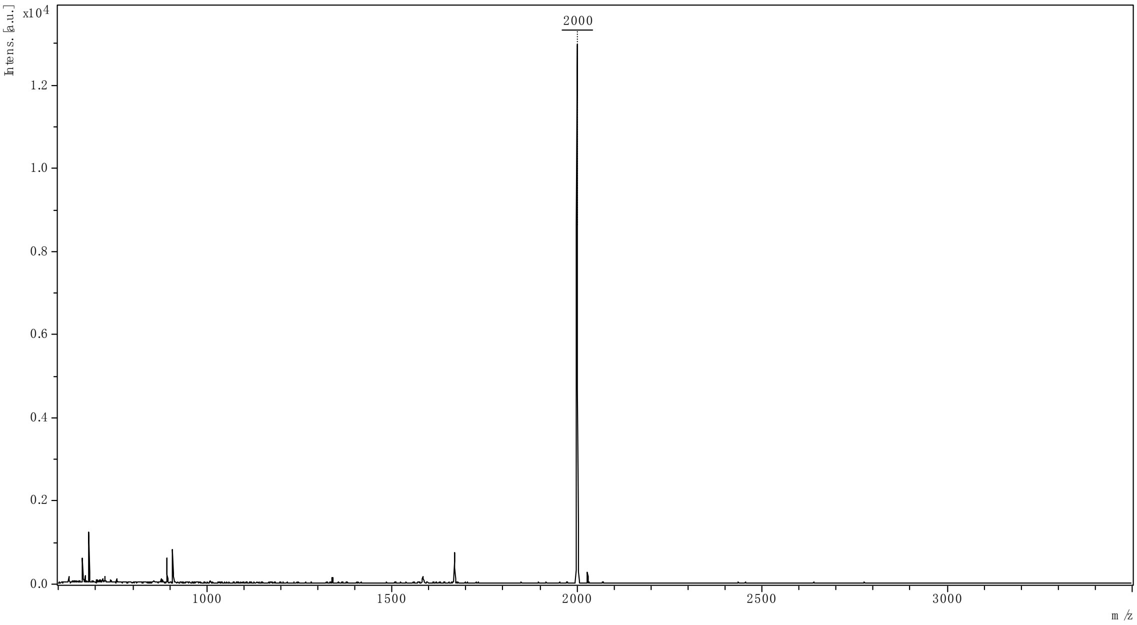

Figure S7. MS (MALDI-TOF) of H_2P m/z: calcd for 2000.9224 u; found [M+H$^+$] 2000.0000 u

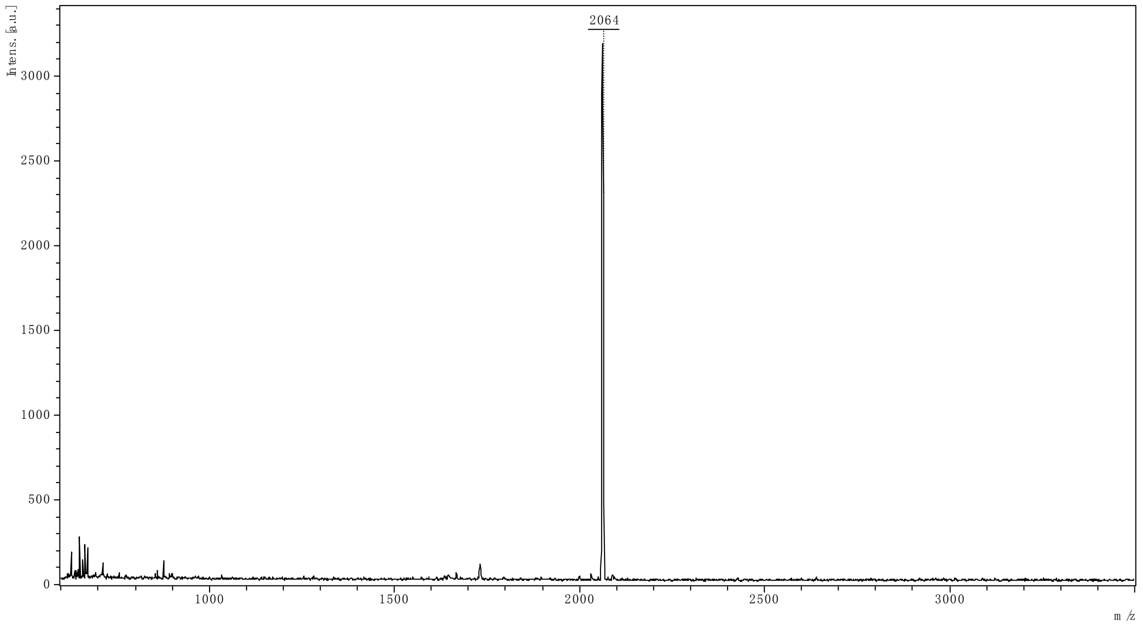

Figure S8. MS (MALDI-TOF) of ZnP m/z: calcd for 2064.2966 u; found [M+H+] 2064.0000 u

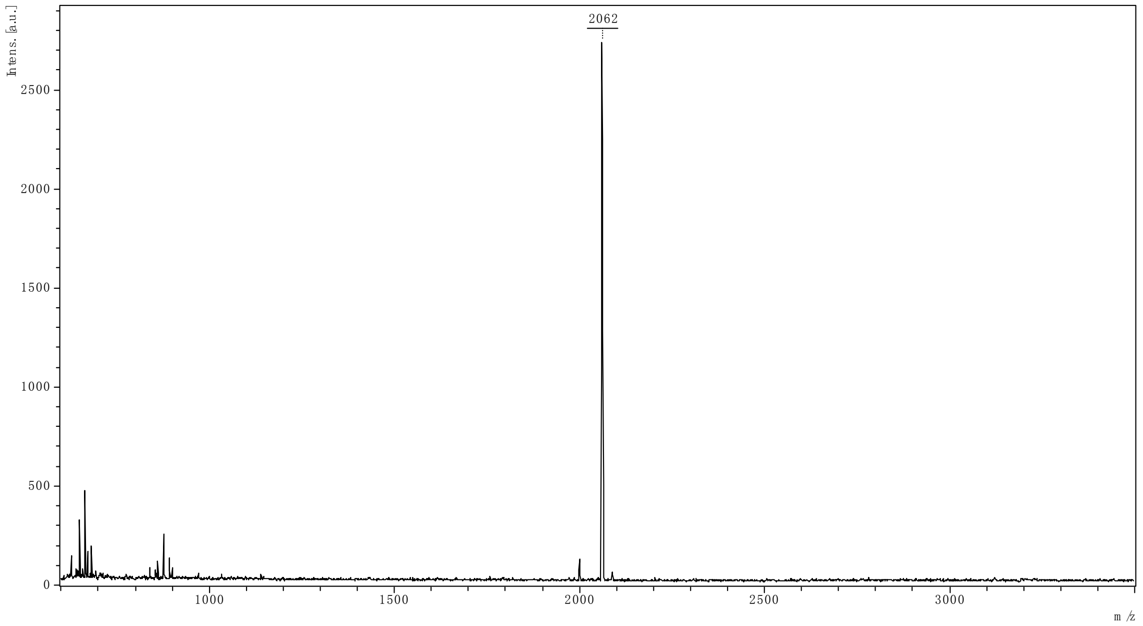

Figure S9. MS (MALDI-TOF) of CuP m/z: calcd for 2062.4526 u; found [M+H+] 2062.0000 u

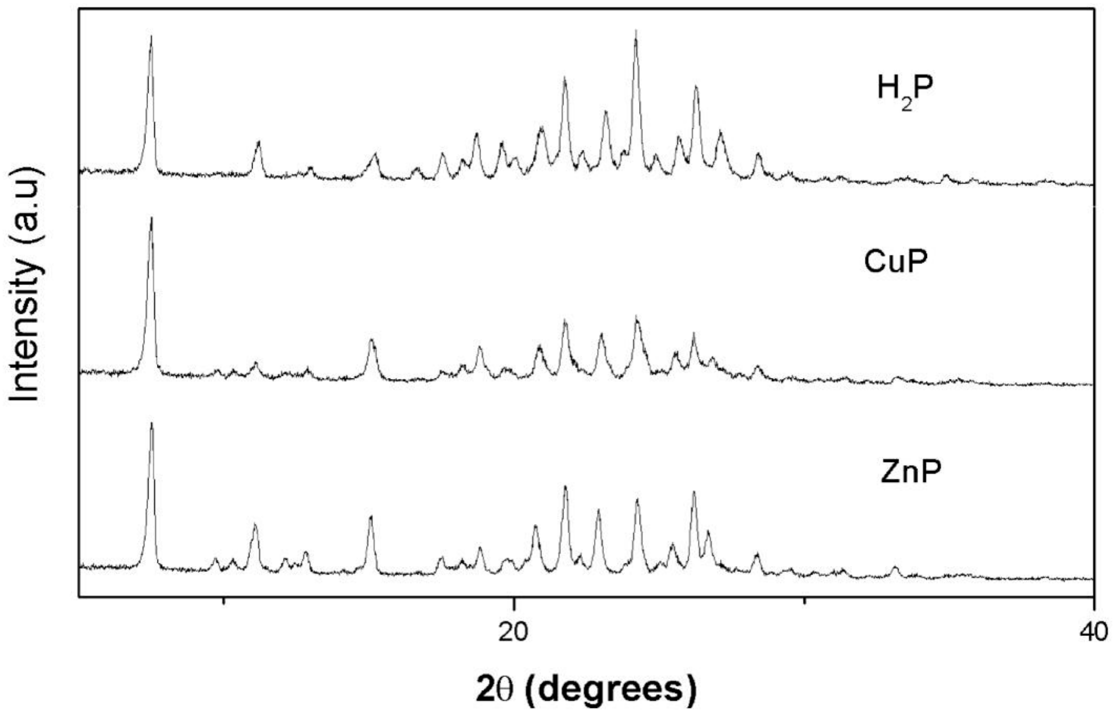

Figure S10. X-ray powder diffraction spectra of CuP, H_2P and ZnP compounds.

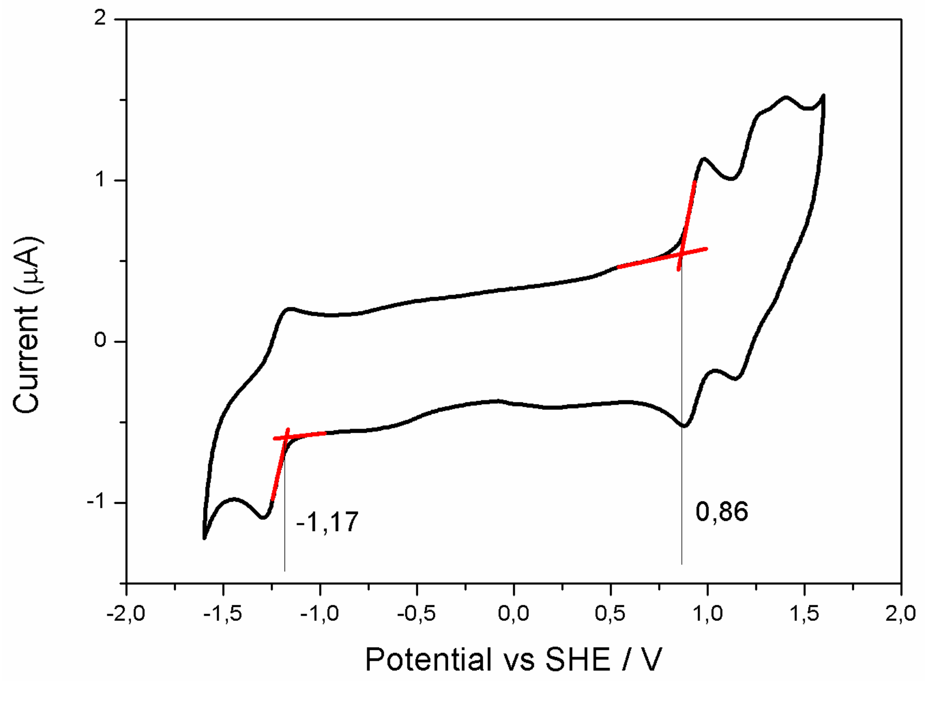

Figure S11. Cyclic voltammogram of H_2P in CH_2Cl_2, 50 mM of $TBAPF_6$. Scan rate = 50 mV/s.

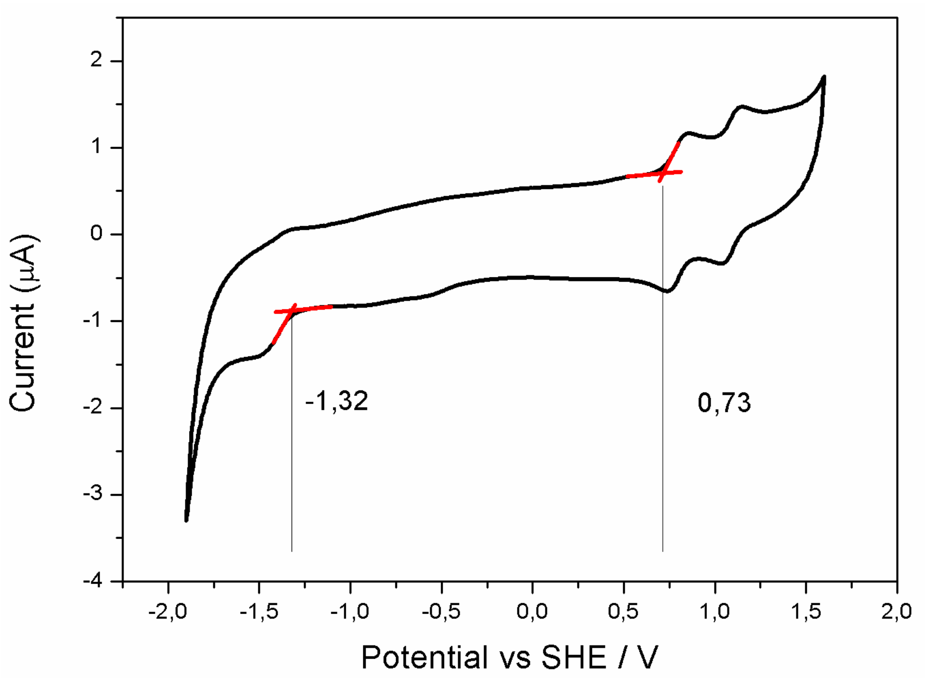

Figure S12. Cyclic voltammogram of ZnP in CH_2Cl_2, 50 mM of $TBAPF_6$. Scan rate = 50 mV/s.

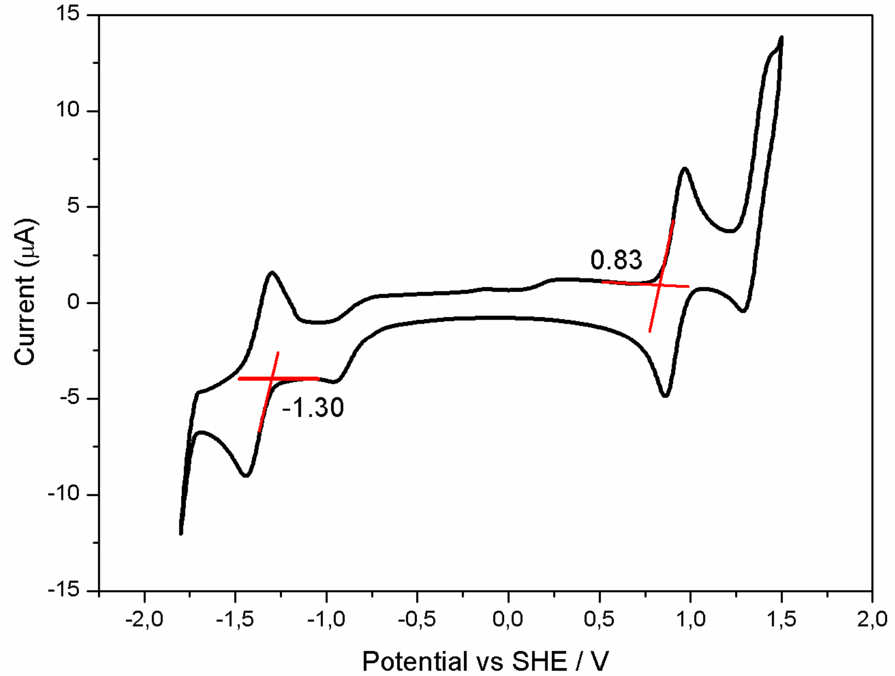

Figure S13. Cyclic voltammogram of CuP in CH_2Cl_2, 50 mM of $TBAPF_6$. Scan rate = 50 mV/s.

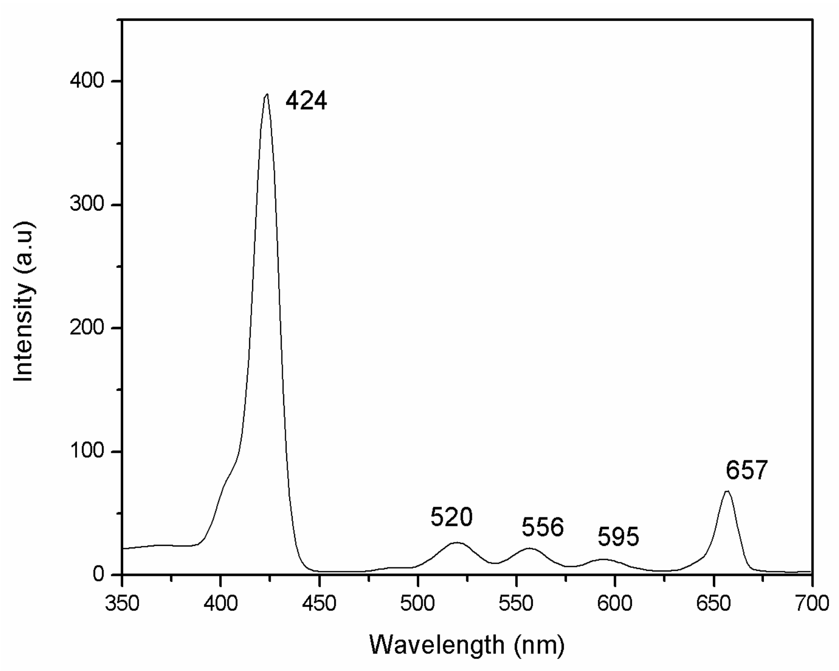

Figure S14. Fluorescence excitation spectrum of H_2P monitored at 657 nm.

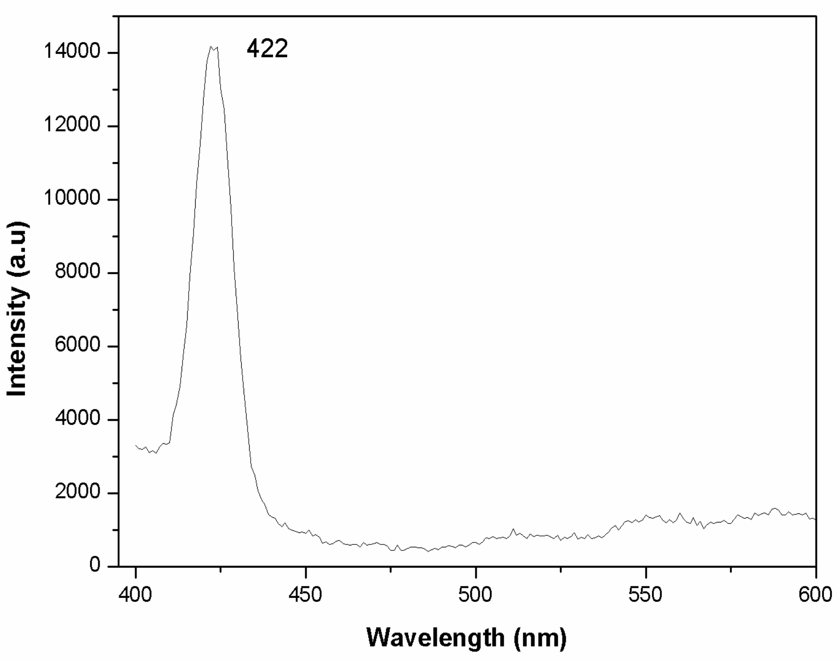

Figure S15. Fluorescence excitation spectrum of ZnP monitored at 601 nm.
